# The Relationship between Depression and Metabolic Syndrome in the Elderly Population: The Cohort Aging Study

**Published:** 2018-10

**Authors:** Afsaneh Bakhtiari, Maryam Hashemi, Seyed Reza Hosseini, Shabnam Omidvar, Ali Bijani, Farzan Khairkhah

**Affiliations:** 1 Social Determinants of Health Research Center, Health Research Institute, Babol University of Medical Sciences, Babol, Iran.; 2 Midwifery Department, Faculty of Medicine, Babol University of Medical Sciences, Babol, Iran.; 3 Departments of Psychiatry, Faculty of Medicine, Babol University of Medical Sciences, Babol, Iran.

**Keywords:** *Amirkola*, *Blood Pressure Component*, *Depression*, *Elderly*, *Metabolic Syndrome*

## Abstract

**Objective:** Metabolic syndrome (MetS) and depression are two important causes of disability in the elderly. The association between MetS and depressive symptoms in Iranian elderly is unclear. In this population-based study, we aimed at evaluating the relationship between MetS and its components with depression in Iranian elderly population.

**Method** **:** This cross sectional study was derived from Amirkola Health and Ageing Project (AHAP).The participants of this study included 1560 elders over the age of 60 during 2012 and 2013. MetS was diagnosed based on Adult Treatment Panel III report and depressive symptoms according to Geriatric Depression Scale. Odds ratio (OR) and 95% confidence interval (CI) based on age and gender were estimated using regression logistic model.

**Results: **Depressive symptoms were observed in 28.7% of men and 46.2% of women. Age- and gender-adjusted OR of depressive symptoms did not show a significant difference among the participants with or without MetS. A significant association between MetS components (including waist circumference, HDL-C, fasting blood glucose, triglyceride) and depressive symptoms was observed, but this association no longer existed after age and gender adjustment. Elevated blood pressure revealed a significant relationship with depressive symptoms in men only (OR, 0.665; 95% CI, 0.469-0.943).

**Conclusion: **Depressive symptoms were associated with blood pressure component but not MetS in the elderly population of Amirkola, Iran. This association highlights the relevance of norepinephrine signal and sympathetic nervous activity disturbance for the emergence of depressive symptoms in the elderly. Therefore, it is reasonable to consider depression in hypertensive patients, especially in men.

All countries are experiencing growth in the number and proportion of the elderly population (1). Increased longevity is associated with an increased risk of aging-associated diseases and an increased chronic disease burden (2). Metabolic syndrome (MetS) and depression are two common chronic disorders among the elderly which can carry undesirable outcomes, including cardiovascular disease (CVD) and disability (3).

MetS is a set of metabolic and hemodynamic abnormalities, including abdominal obesity, impaired lipid profile and glycemic control (4). 

The International Diabetes Federation estimates that nearly 25% of the world's population has MetS (5). Similar studies among Iranian populations have found comparable prevalence, ranging from 31% to 49.5% of the studied adults, with greater prevalence in the elderly (6, 7). It has been documented that MetS complications are exacerbated by aging and physiological changes (8).

Depression is also another common disorder in aging.

Prevalence of depressive disorders among the elderly varies from 10% to 20%, depending on the demographic and social characteristics (WHO). 

A recent meta-analysis of the prevalence of depression in the Iranian elderly was reported to be 43% (9). Depression in the elderly leads to poorer health, slower recovery, more physical pain, higher health care costs, and lower quality of life and also increased risk of stroke, heart failure, hip fractures, and death rates from other illnesses (10). Effective prevention of the burdens associated with depression requires a strategy based on a better knowledge of the related risk factors. MetS might accelerate depressive symptoms in the elderly because of the correlation between depression and non-communicable chronic diseases, such as diabetes, blood glucose, and dyslipidemia and poor control of these diseases by depressed people (11).

The relationship between MetS and psychological problems yielded conflicting results (2-5), either pointing to no relationship (12, 13), a relationship only between certain components of MetS with depression (14, 15), a gender-specific association (11, 15), or a relationship between the two variables (16, 17). MetS and depression are two important issues in the elderly's health field. Therefore, given the ambiguity in the relationship between MetS and depression in previous studies as well as the failure of conducting research in Iran, especially among the elderly in a large population-based study, this study was conducted to determine the relationship between MetS and its components with depressive symptoms. The study results can be helpful in the elderly care intervention programs.

## Materials and Methods

***Study Population***

Amirkola Health and Ageing Project (AHAP) is a prospective cohort study of social determinants of health in an urban community of Amirkola in north of Iran. Based on data from the national census, the population of the city is 26,232, with 2234 people aged 60 years or older. All the elderly residents of Amirkola were invited to participate in this cross sectional study during 2011 and 2012. A total of 1616 elders participated in this study. After excluding 56 participants due to missing data on depression or MetS, 1560 remained in the study. Details of the procedure were published in a previous article (18). The study protocol was approved by the Ethics Committee of Babol University of Medical Sciences (MUBABOL.REC.1392.14). All participants provided an informed written consent form. 

***Measurements of Metabolic Syndrome***

The MetS was defined according to the National Cholesterol Education Program’s Adult Treatment Panel III report (ATP III) criteria. MetS was diagnosed when a person had three or more of the following components: waist circumference (WC) > 88cm for women and > 102 for men, systolic blood pressure ≥130 mmHg or diastolic blood pressure ≥ 80 mmHg, high-density lipoprotein- cholesterol (HDL -C) < 40 in men and < 50 in women, triglyceride (TG) ≥150, fasting blood glucose (FBG) ≥100 mg/dL, or being treated for low HDL-C or high blood pressure, TG, and FBG. 

In the study, the diagnosis of hypertension was based on blood pressure measurement using the Omron manometer M3 intelligence model in lying position at two times using standard methods. Average values of these two measurements were considered as hypertension. Weight was measured using Seca digital scale with minimum clothes, with an accuracy of 0.1 kg, and height was measured by stadiometer with error of 0.5 cm. To measure blood glucose and lipids, after a 12-hour overnight fast, venous blood was drawn and collected in the tubes containing ethylene diamine tetra acetic acid to obtain centrifuged plasma. 

***Depression Scale***

Information related to depressive symptoms was collected using Geriatric Depression Scale (GDS).The reliability and validity of GDS has been assessed in Iran (19, 20). GDS questionnaire contains 15 questions and each item has one score. Questions 1, 5, 7, 11, and 13 are considered as depression points in the event of the negative response; the 10 remaining questions are calculated based on the scores in the event of positive answer. Based on this procedure, the patients were divided into 4 groups: 0-4 = normal, 5-8 = mild, 9-11 = moderate and 12-15 = severe depression. Elderly patients with a score ≥5 were in need to be referred to a psychiatric clinic and have a clinical interview with a psychiatrist for diagnosis of depression. Thus, the participants were divided into two groups of depressed and non-depressed; then, the two groups were evaluated for the presence of MetS. Sensitivity of this test was 92% and its specificity was 89%. The Cronbach's alpha of the questionnaire was 0.81 in the elderly population of Amirkola.

In addition, for each sample, a demographic questionnaire was completed, which included age, job, education, marital status, and smoking habit. 

***Statistical Analysis***

In the present analysis, the key risk factor was MetS and its components, and the outcome was depressive symptoms. MetS components were classified as normal or abnormal according to the definition of ATP III. The prevalence of depression in each subgroup of MetS components was analyzed based on gender.

The association between MetS and its components with depression was evaluated by independent t test. The association between components of Mets and depressive symptoms was evaluated in two stages. In the first stage, an age and gender-adjusted logistic regression model was used, and gender-split was used in the second stage. Statistical analysis was performed using SPSS 20 software. P <0.05 was considered significant.

## Results

***Characteristic of the Study Population***

The samples included 699 women and 861 men (n = 1560), with the mean age of 69.3±7.4 and ranged between 60 and 92 years. With respect to educational level, 64% were illiterate, 29.3% had middle and high school education, and 6.7% had high school diploma or higher. Of the participants, 85.3% were married and 31.5% were employed (compared to 68.5% unemployed). Table 1 displays clinical features of the participants.

***Association of Metabolic Syndrome with Depression***

The overall prevalence of MetS was 71%, with higher prevalence in women than in men (89% vs. 57%; p<0.001). Among the individuals with MetS, 213 (13.7%) had all five components of MetS in which the most common component was low levels of HDL-C (83.7%), followed by high blood pressure (77.4%), high FBG (57.4%), abdominal obesity (52.6%), and high triglycerides (44%) (Table 1). Depressive symptoms were observed in 665 of the participants (42.6%), including 418 women (59.8%) and 247 men (28.7) (<0.001). Frequency distribution of MetS components indicated that the amounts of FBG and TG are significantly higher among individuals with depressive symptoms compared to healthy individuals (p<0.001, p<0.01) (Table 2). 

Table 3 demonstrates crude age and gender adjusted odd ratio (OR) for depressive symptoms according to the components of MetS. Components of MetS were classified as normal or abnormal according to ATP III definition. 

The crude OR showed higher prevalence of depressive symptoms among individuals with MetS (OR = 1.69, 95% CI = 1.34-2.13) and also in individuals with abnormal levels of TG, HDL-C, FBG, and WC. This association disappeared after age and gender adjustment.

Table 4 demonstrates the association between MetS and depressive symptoms among men and women. MetS and its components were not predictive factors for depressive symptoms in either men or women, except for high blood pressure, which showed a significant association with higher prevalence of depressive symptoms among men only (OR = 0.66, 95% CI = 0.47-0.94). This association remained even after age adjustment (OR = 0.67, 95% CI = 0.48-0.95). 

## Discussion

The results of this population-based study showed no association between the MetS and its components with depressive symptoms, except for high blood pressure, which showed a significant association with higher prevalence of depressive symptoms among men even after age adjustment.

To our knowledge, this was the first study to investigate the relationship between depressive symptoms and MetS in Iran, which was performed in a large group of homogenous elderly population with a high prevalence of MetS and depressive symptoms, especially in women.

This result is consistent with two of the largest studies, to date, in Norway and Finland. The first study was conducted on 9571 participants aged 20-89 years. The researchers found no association between anxiety and depression with MetS despite the generous statistical power and use of both continuous and categorical approaches (13). Another study on 5698 young participants also obtained similar results (12).

With regards to cross sectional studies, a study in Croatia (2013) on 203 individuals showed lack of relationship between MetS components in depressed patients (21). Another study in Italy (2011) on depressed patients over 65 years showed that depressive symptoms also starts by an increase in WC in older ages and that the MetS alone is not a factor for developing depression but may increase the persistence of depression (22). A cohort study from France demonstrated no significant association between MetS and depressive symptoms in the elderly aged 70–90 years (23). With regards to Asian population, a study (2011) on 458 Japanese men and women aged 21-87 years showed no relationship between MetS and depression in men and women; however, only a weak correlation was observed between the increase in FBG and depression in men (15). Another study in Japan (2009) was conducted on 956 men and only found a relationship between increased WC and depression (14). A cross sectional study in 200 elderly patients hospitalized with cardiovascular disease found no significant relationship between MetS and depression in Iran. Among the MetS components, only high systolic blood pressure was significantly associated with depression (24).

However, some studies suggest a relationship between mood disorders and MetS. Hung et al. (2014) study on 56 bipolar patients showed that bipolar disease has a very strong relationship with MetS. Antipsychotic medicines also increase lipid disorders and MetS (25).This study referred to a specific psychiatric illness called bipolar disease that can be a feature of mental illness, but it is independent of depression. Another study in France (2007) showed high depressive symptoms in men and women with MetS but no relationship with anxiety (16). A study on 1386 Japanese male workers (2011) showed that the incidence of MetS was significantly higher in patients with an increase in depressive symptoms than those without depression (17). In explaining the relationship between MetS and depression, Ohmori et al. (2017) proposed lifestyle role (26). Their findings revealed that depression is associated with several unhealthy behavioral factors in both men and women, but depression was associated with Mets only in women. These findings suggest that depression may be a warning sign of MetS in women with unhealthy behaviors. The researchers concluded that further investigations are needed to clarify the underlying causes of association between MetS and depression.

Regarding the physiological mechanism of the incidence of psychological disorders in MetS, some have suggested that mental health problems are associated with the mechanism of accumulation of visceral fat to metabolic disorders (12). Another mechanism is dysregulation hypothesis of chronic sympathetic and neuroendocrine system. This hypothesis suggests that stress and unfavorable social conditions, from personal point of view, act as triggering factors, which may lead to metabolic and anthropometric changes over time for the MetS characteristics. This concept occurs through hemodynamic and neuroendocrine reactions (27, 28). Other studies also showed HPA-axis (hypothalamic-pituitary-adrenal) stimulation due to stress caused by depression. The increased activity of HPA-axis leads to increased androgens and changes in secretion of sexual steroids and growth hormones, which can also cause metabolic changes. They believe that the hyperactivity of the HPA-axis, which is common in depression, can lead to metabolic changes (29). Also, behavioral factors associated with depression symptoms, such as physical inactivity and poor diet, may affect obesity and metabolic disorders in the body of elderly population. The present study did not have data to examine any of these hypotheses. Additional research is needed to examine these and other potential pathways.

The difference in the results of the relationship between MetS and depression can be due to study design, use of different psychological scales, and different diagnostic criteria for MetS in various studies. Study groups also have an important role in these differences. In most studies, samples were not the general population with MetS; they were rather young or only men or women before menopause or a clinically specific target population. However, considering that our study tried to evaluate depressive symptoms and MetS simultaneously and given the large number of samples, the homogeneity of the study population, and use of ATP III criteria for MetS as one of the best methods used in MetS research and adopted Iranian cut-off point values, this research can provide reliable results in this regard.

In our study, although the number of components of MetS was higher in women than men, the influence of gender on the relationship between depression and MetS was not statistically significant. However, in some studies, the relationship between MetS and depression was significant in only one gender. Gil et al. study (2006) in Poland found a significant relationship between the MetS and depression in women (30). Also, a study in Korea (2014) showed that the relationship between MetS and depression was significant only in women (25). Previous studies in Italy (2009) on 353 individuals over 75 years (31) and in the United States (2004) on 186 men and 300 women aged 17 to 39 years showed that this relationship was significant only in women (32). However, in the study of Sekita et al. (2013) in Japan, the only significant relationship was found in men (3).

The difference may stem from genetic, hormonal, social and economic factors, and different social roles between men and women. Increased activity of the HPA axis resulted from depression can reduce estrogen levels, which eventually leads to menopause and increased visceral fat, and this can be one of the causes of MetS in women. 

In the present study, among the MetS components, only high blood pressure was associated with depressive symptoms in men, when analyzed as binary measure (yes, no) by logistic regression models even after adjusting for age. The pathways through which depression could increase the risk of subsequent hypertension remain to be determined. However, proposed possible mechanisms include the increased plasma norepinephrine, arousal and mobilization of energy stores as well as alterations of serotonin-mediated platelet activation described in affective disorders (33, 34). A meta-analysis of prospective cohort studies (2012) showed that both depressive and hypertensive patients experience increased sympathetic tone and increased secretion of adrenocorticotropic hormone and cortisol (35). Therefore, it is pathophysiologically plausible that depression and hypertension affect each other. Lambert et al. (2010) in Australia showed a relationship between sympathetic activity and increased blood pressure and the mood problems with MetS (36). Similarly, Rubio-Guerra and et al. (2013) found a high prevalence of depression in hypertensive patients; this prevalence was approximately nine times greater than what is observed in the general population (27). However, there is skepticism over the findings which indicated an association between depression and hypertension (36, 37) although recent follow-up studies depicted that progression of depression would predict hypertension later in life (38, 39). It is unknown why blood pressure was high only in depressed men, but one of the reasons could be related to demographic determinants of hypertension disease. For example, Balog et al. (2013) found that marital distress was independently associated with hypertension treatment only in men (40). However, more research is needed to clarify this relationship.

Our study was conducted to evaluate depressive symptoms and MetS simultaneously. The strength of the present study was that the AHAP study includes the whole elderly population of Amirkola, Iran, with the participation rate of 70%, homogeneity of the study population, and their great cooperation. This study also used information from the first phase of the Amirkola cohort study (a community-based study) that relates to just one section of the study. We hope to achieve more valuable results in future studies using the data from the second phase of this cohort (41).

**Table 1:** Clinical Characteristics of Metabolic Syndrome of Amirkola Elderly Population (N = 1560)

**Variables**	**Ferequency**	**Percentage**
Intensity of depressive symptoms		
Normal	895	57.4
Mild	420	26.9
Moderate	169	10.8
Severe	76	4.9
Individual components of MetS		
WC	821	52.6
Blood pressure^a^	1208	77.4
TG	686	44
HDL-C	1306	83.7
FBG	896	57.4
Number of MetS Components		
0	11	0.7
1	134	8.6
2	336	21.5
3	418	26.8
4	448	28.7
5	213	13.7
	Mean ± SD	Min-Max
Age (years)	69.3 ± 7.4	60-92
GDS (score)	4.5 ± 3.5	0-14
WC (cm)	95.7 ± 10.5	50-128
SBP (mm/hg)	142.7 ± 22.2	85-229
DBP (mm/hg)	81.5 ± 119	43-142
FBG (mg/dl)	118 ± 45.7	60-458
TG (mg/dl)	160.3 ± 84	52-812
HDL-C (mg/dl)	38.7 ± 4.4	30-68

Note: WC: waist circumference; SBP: systolic blood pressure; DBP: diastolic blood pressure; FBG: fasting blood glucose; TG: triglyceride; HDL-C: high density lipoprotein; GDS: geriatric depression scale; MetS: metabolic syndrome.

a Defined as high systolic or diastolic blood pressure or current drug therapy for known hypertension

**Table 2:** Frequency Distribution of the Components of Metabolic Syndrome, Age, and Gender in the Elderly with and without Depressive Symptoms^a^

**Variables**	**Depressive Symptoms**	**Normal**	**P value**
	**N==665**	**N= 895**	
Gender Male Female	247 (28.7) 418 (59.8)	614 (71.3) 281 (40.2)	0.001
Age (years)	69.5±7.3	69.1±7.4	0.31
WC (cm)	95.8±10.5	95.7±10.5	0.86
SBP (mm/hg)	142.1±23	143.2±21.6	0.32
DBP (mm/hg)	81.4±12.6	81.5±113	0.84
FBG (mg/dl)	122±50.2	115±41.7	0.003
TG (mg/dl)	166.3±89.7	155.9±79.4	0.01
HDL-C(mg/dl)	38.9±4.6	38.6±4.2	0.13

Note: WC: waist circumference; SBP: systolic blood pressure; DBP: diastolic blood pressure; FBG: fasting blood glucose; TG: triglyceride; HDL-C: high density lipoprotein.

a Values shown are means±SD or numbers of participants (percentages).

**Table3:** The Association between Metabolic Syndrome and Its Components among the Elderly with and without Depressive Symptoms

**Variables**	**Depressive Symptoms** a	**Normal** b	**Crude OR (95%, CI)**	**P-value**	**Adjusted OR** 1 ** (95%, CI)**
MetS yes no	518 (46.2) 147 (33.6)	604 (53.8) 291 (66.4)	1.69 (1.34-2.13)	0.001	1.03 (0.94-1.14)
WC Abnormal Normal	403 (50.7) 262 (34.2)	392 (49.3) 503 (65.8)	1.97 (1.60-2.42)	0.001	1.03 (0.79-1.32)
BP^2^ Abnormal Normal	540 (57.2) 125 (41.9)	722 (42.8) 173 (58.1)	1.03 (0.80-1.33)	0.84	0.92 (0.70-1.21)
FBG Abnormal Normal	412 (44.8) 253 (39.5)	507 (55.2) 388 (60.5)	1.20 (1.01-1.53)	0.03	1.11 (0.89-1.39)
TG Abnormal Normal	375 (45.3) 290 (39.6)	452 (54.7) 443 (60.4)	1.26 (1.04-1.55)	0.02	1.03 (0.82-1.27)
HDL-C Abnormal Normal	596 (45.3) 69 (28.3)	720 (54.7) 175 (71.7)	2.09 (1.55-2.83)	0.001	1.17 (0.85-1.62)

Note: WC: waist circumference; BP: blood pressure; FBG: fasting blood glucose; TG: triglyceride; HDL-C: high density lipoprotein.

1 Adjusted for age and gender.

2 Blood pressure was defined as high systolic or diastolic blood pressure or current drug therapy for known hypertension.

a,b Values shown are numbers of participants (percentages).

**Table 4:** The Association between Metabolic Syndrome and Its Components with Depressive Symptoms among the Elderly According to the Gender

**Variables**		**Male**		**P-value**		**Female**		**p-value**
	**Depressive ** **Symptoms** a	**Crude OR** **(95%, CI)**	**Adjusted OR** 1 **(95%, CI)**		**Depressive ** **Symptoms** a	**Crude OR** **(95%, CI)**	**Adjusted OR** 1 **(95%, CI)**	
MetS Yes No	135 (27.3) 112 (30.5)	0.86 (0.64-1.15)	0.98 (0.86-1.11)	0.31	383 (61) 35 (49.3)	1.61 (0.98-2.63	1.12 (0.96-1.29)	0.06
WC Abnormal Normal	65 (28) 182 (28.9)	0.96 (0.68-1.33)	0.97 (0.99-1.03)	0.79	338 (60) 80 (58.8)	1.05 (0.72-1.53)	1.01 (0.75-1.62)	0.84
BP Abnormal Normal	183 (26.9) 64 (35.4)	0.67 (0.48-0.95)	0.66 (0.47-0.94)	0.02	357 (61.3) 61 (52.1)	1.45 (0.98-2.17)	1.40 (0.94-2.09)	0.07
FBG Abnormal Normal	140 (30) 107 (27.1)	1.16 (0.86-1.55)	1.18 (0.87-1.69)	0.34	272 (60) 146 (59.3)	1.02 (0.75-1.41)	1.05 (0.76-1.43)	0.86
TG Abnormal Normal	104 (27.2) 143 (29.9)	0.88 (0.65-1.18)	0.90 (0.67-1.22)	0.39	271 (60.90 174 (39.1)	1.13 (0.83-1.55)	1.16 (0.85-1.59)	0.43
HDL-C Abnormal Normal	186 (29.4) 61 (26.6)	1.15 (0.82-1.61)	1.15 (0.82-1.62)	0.44	410 (59.9) 8 (53.3)	1.30 (0.46-3.65)	1.34 (0.48-3.75)	0.60

Note: WC: waist circumference; BP: blood pressure; FBG: fasting blood glucose; TG: triglyceride; HDL-C: high density lipoprotein.

1 Adjusted for age

a Values shown are numbers of participants (percentages).

## Limitation

This study had some limitations. First, determining a causal correlation between MetS and depressive symptoms progression was unclear due to the cross-sectional nature of the study. In addition, we were unable to address the potential mechanisms underlying the reported association between blood pressure and depression in men.

## Conclusion

This study showed no association between MetS and its components with depressive symptoms in a sample of elderly population. However, hypertension was associated with depressive symptoms only in men. Access to a large, population-based cohort database validates our results on this putative association in an elderly population. We suggest that, in future studies, these associations be investigated in large longitudinal population-based cohorts to better highlight the putative association of blood pressure with depression.
